# Supplementary material for: Molecular Insight into the Regulation of Vimentin by Cysteine Modifications and Zinc Binding
Source: Antioxidants (Basel). 2021 Jun 28;10(7):1039. doi: 10.3390/antiox10071039 (PMC8300659; doi:10.3390/antiox10071039)
Supplement: Supplementary file 1 [file antioxidants-10-01039-s001.zip › antioxidants-1250357-supplementary.pdf]

Supporting Information

Molecular insight into the regulation of vimentin by cysteine modifications and zinc binding

Andreia Mónico, Joan Guzmán-Caldentey, María A. Pajares, Sonsoles Martín-Santamaría, Dolores Pérez-Sala

Department of Structural and Chemical Biology, Centro de Investigaciones Biológicas Margarita Salas (CSIC), Ramiro de Maeztu, 9. 28040 Madrid, Spain.

Table of Contents

Figure S1. MD simulations of vimentin dimer A (100 ns) and vimentin dimer B (200 ns). RMSD plots.

Figure S2. MD simulation of vimentin dimer B with Zinc ions (30 ns). RMSD plot.

Figure S3. MD simulation of vimentin dimer B with Cys328 thiol group as thiolate and in presence of Zinc ions (50 ns). RMSD plot.

Figure S4. Determination of Cys328 pKa through various methods.

Figure S5. Competition assay between vimentin and Zincon for zinc binding.

Figure S6. MD simulation of vimentin dimer Cys328Ser mutant (50 ns). RMSD plot.

Figure S7. MD simulations of vimentin tetrameric complexes (100 ns). A) "distant-CYS" (Cys residues at a distance of 22 Å). B) "nearby-CYS" (Cys residues at a distance of 4 Å). RMSD plots.

Figure S8. MD simulation of vimentin tetrameric "nearby-CYS" complex with Zinc ions (100 ns). RMSD plot.

Figure S9. MD simulation of vimentin tetrameric Cys328Ser mutant complex (100 ns). RMSD plot.

Figure S10. Protective effect of zinc on Cys328 modification and crosslinking in soluble and polymerized vimentin

Figure S11. Cysteine crosslinkers used in this study.

Figure S12. MD simulation of vimentin dimer B with Magnesium ions (100 ns). RMSD plot.

Figure S13. Effect of millimolar magnesium concentration on the crosslinking of vimentin by DBB.

Supplementary Figures

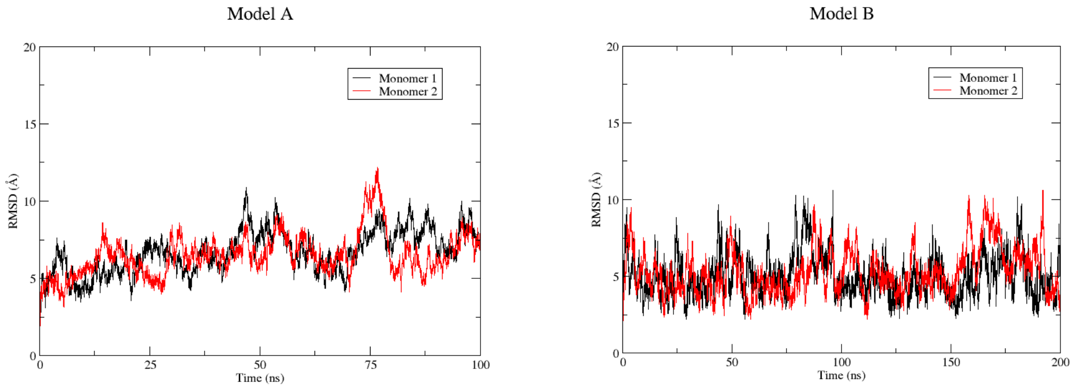

Figure S1. MD simulations of vimentin dimer A (100 ns) and vimentin dimer B (200 ns). RMSD plots for the α-Carbons.

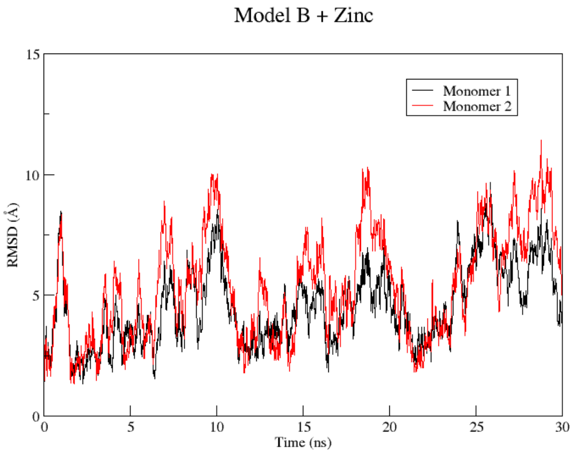

Figure S2. MD simulation of vimentin dimer B with Zinc ions (30 ns). RMSD plot for the α-Carbons.

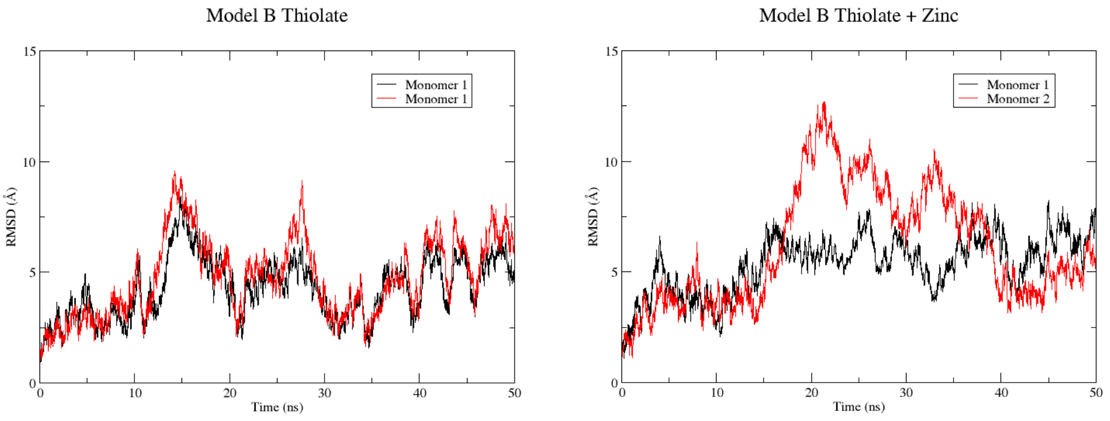

Figure S3. MD simulation of vimentin dimer B with Cys328 thiol group as thiolate and in presence of Zinc ions (50 ns). RMSD plot for the α-Carbons.

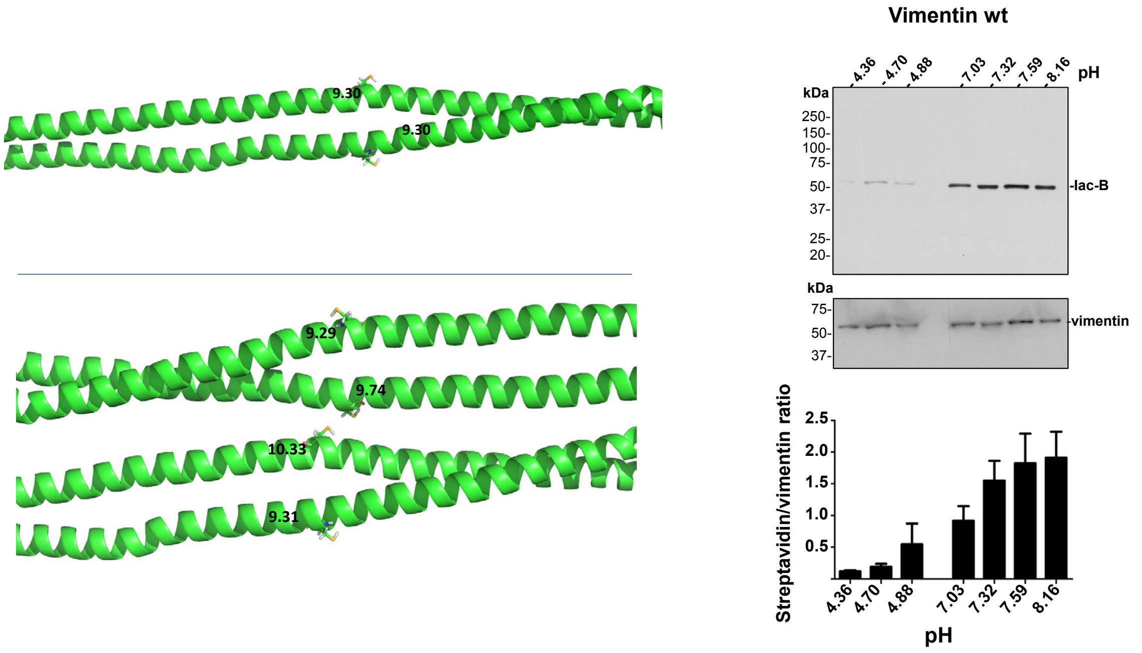

Figure S4. Determination of Cys328 pKa through various methods. Computational methods were used to calculate C328S, as detailed in Methods in the context of the vimentin dimer or Nearby CYS tetrameric complex (left panels). Right panel, experimental determination of Cys328Ser pKa through the incorporation of biotinylated iodoacetamide. Western blot analysis of vimentin wt (0.8 μg) modification with biotinylated-iodoacetamide (Iac-B) at pH ranges around the pKa values measured with monobromobimane (please see main text). Biotin incorporation was estimated using

streptavidin-HRP (upper blot) and vimentin levels were estimated by western blot with anti-vimentin V9 antibody (lower blot). Densitometric scanning was performed with ImageJ and the streptavidin/vimentin signal ratio obtained as shown in the histogram. The figure shows the mean ± SEM of three independent experiments.

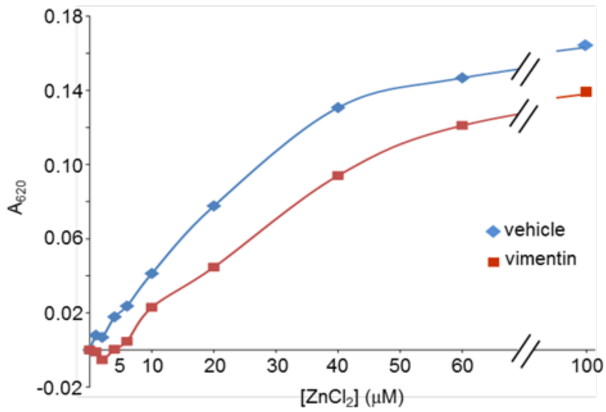

Figure S5. Competition assay between vimentin and Zincon for zinc binding. Vimentin at 5 µM or vehicle (detailed in Materials and Methods) were added to mixtures containing 40 µM Zincon and the indicated concentrations of $ZnCl_2$. Formation of the Zincon-Zn^{2+} complex was assessed by measuring absorbance at 620 nm. The figure shows one representative experiment out of three carried out with similar results.

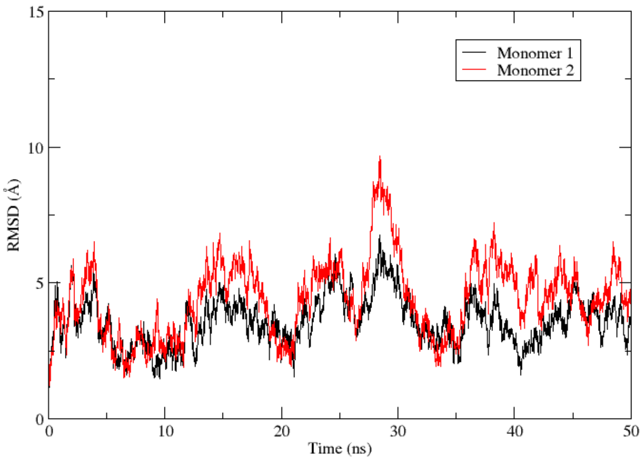

Figure S6. MD simulation of vimentin dimer Cys328Ser mutant (50 ns). RMSD plot for the α-Carbons.

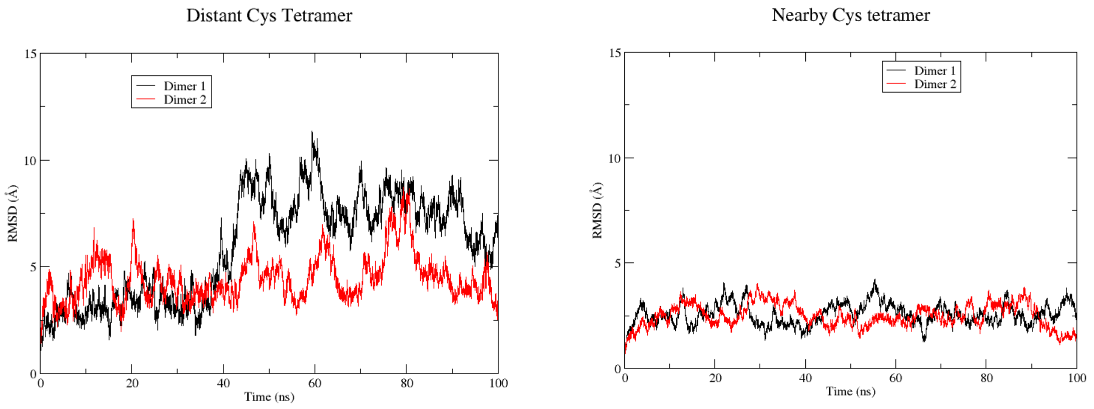

Figure S7. MD simulations of vimentin tetrameric complexes (100 ns). A) "distant-CYS" (Cys residues at a distance of 22 Å). B) "nearby-CYS" (Cys residues at a distance of 4 Å). RMSD plots for the α-Carbons.

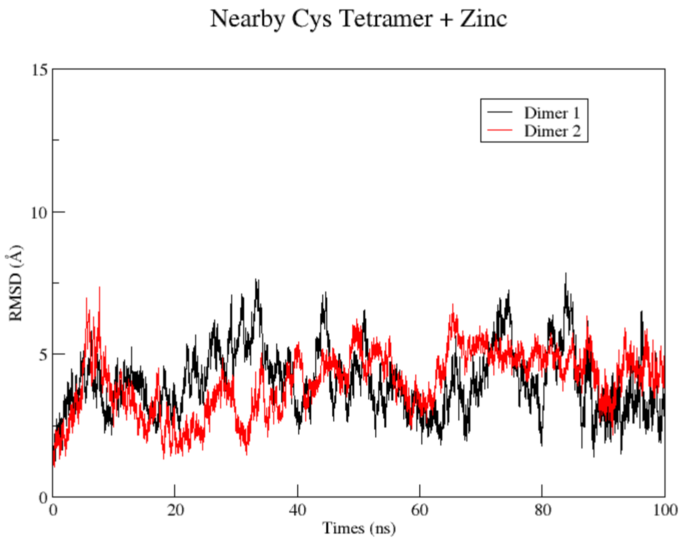

Figure S8. MD simulation of vimentin tetrameric "nearby-CYS" complex with Zinc ions (100 ns). RMSD plot for the α-Carbons.

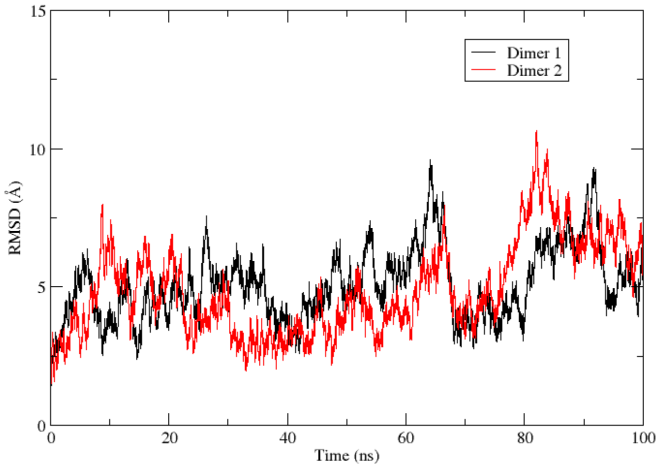

Figure S9. MD simulation of vimentin tetrameric Cys328Ser mutant complex (100 ns). RMSD plot.

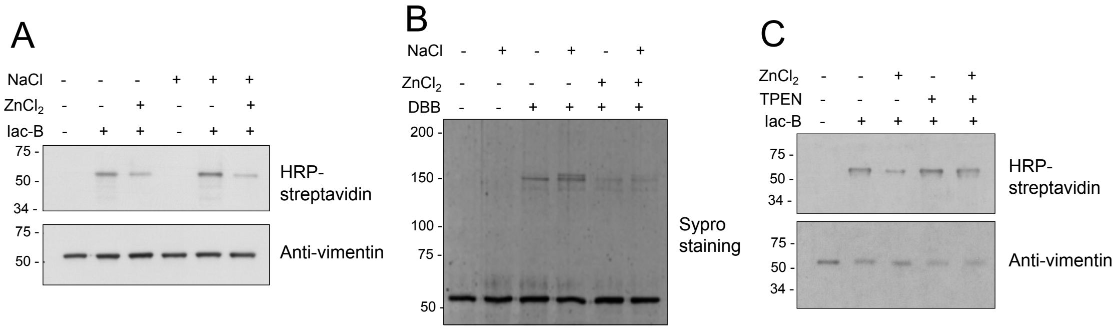

Figure S10. Protective effect of zinc on Cys328 modification and crosslinking in soluble and polymerized vimentin. (A) Vimentin at 5 µM was incubated with vehicle or 150 mM NaCl for 10 min at 37ºC, followed by an 1 h incubation with 500 µM $ZnCl_2$, after which, 10 µM biotinylated iodoacetamide (Iac-B) was added for an additional hour at 37ºC. Samples were analyzed by SDS-PAGE and electroblotted. Incorporation of biotin was assessed by detection with HRP-streptavidin and vimentin levels by western blot. (B) Vimentin was incubated in the absence or presence of NaCl followed by $ZnCl_2$ as in (A) and subsequently treated with vehicle or 24 µM DBB for 1 h t r.t. Samples were analyzed by SDS-PAGE and Sypro-Ruby staining. (C) Vimentin was incubated in the presence or absence of $ZnCl_2$, after which the zinc chelator N,N,N′,N′-tetrakis(2-pyridylmethyl)ethylenediamine (TPEN) was added at 1 mM for 30 min before treatment with Iac-B. Incorporation of biotin was detected as in (A). Results are representative from (A) tree, (B) two and (C) two assays.

Compound	Structure	Spacer (Å)
Dibromobimane (DBB)	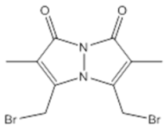	4.9
Tris(2-maleimidoethyl)amine (TMEA)	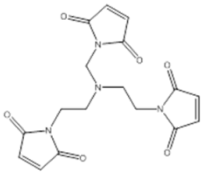	10.3
Bismaleimidohexane (BMH)	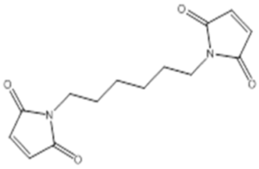	13.0

Figure S11. Cysteine crosslinkers used in this study. Structures of three cysteine-crosslinkers, dibromobimane (DBB), tris(2-maleimidoethyl)amine (TMEA) and bismaleimidohexane (BMH), with the corresponding space arm lengths as specified by the manufacturer.

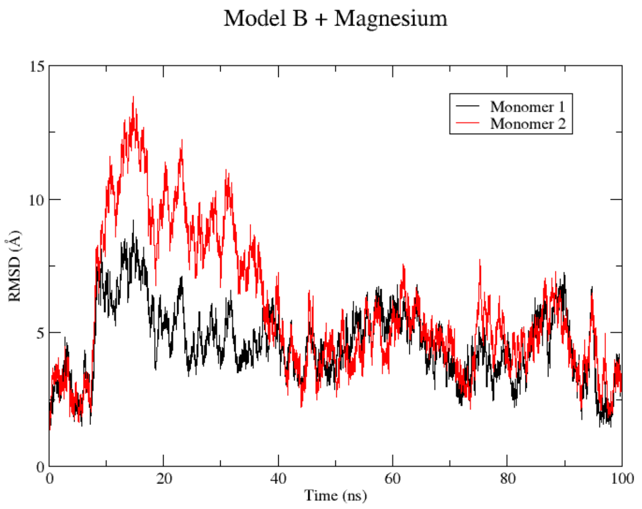

Figure S12. MD simulation of vimentin dimer B with Magnesium ions (100 ns). RMSD plot for the α-Carbons.

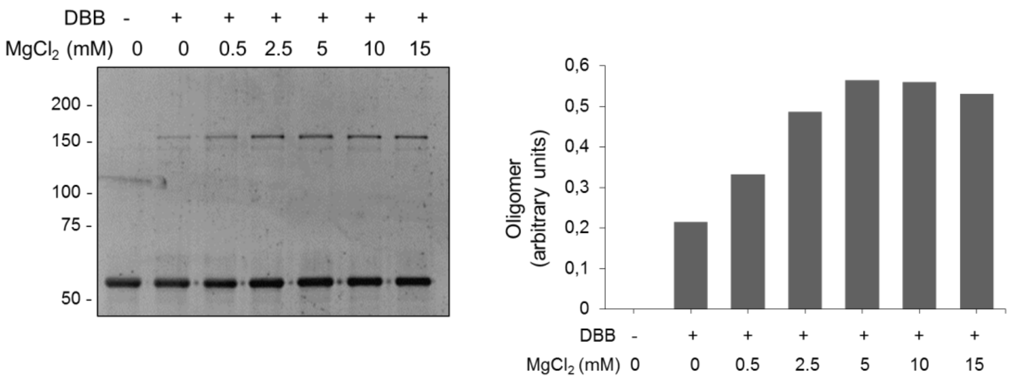

Figure S13. Effect of millimolar magnesium concentration on the crosslinking of vimentin by DBB. Vimentin wt at 4.3 μM was first incubated with the indicated concentrations of $MgCl_2$ for 1 h, at r.t, after which, it was incubated with DBB at 24 μM for 1 h more. Incubation mixtures were analysed by SDS-PAGE and protein species visualized by under UV light after SYPRO Ruby staining. Levels of oligomer were estimated by image scanning and are shown in the histogram at the right.
